# Impairment of Anti-Aggregatory Responses to Nitric Oxide and Prostacyclin: Mechanisms and Clinical Implications in Cardiovascular Disease

**DOI:** 10.3390/ijms23031042

**Published:** 2022-01-18

**Authors:** Yuliy Y. Chirkov, Thanh H. Nguyen, John D. Horowitz

**Affiliations:** Cardiology Laboratory, Basil Hetzel Institute, The Queen Elizabeth Hospital, The University of Adelaide, Adelaide 5011, Australia; yuliy.chirkov@adelaide.edu.au (Y.Y.C.); thanh.h.nguyen@adelaide.edu.au (T.H.N.)

**Keywords:** platelets, nitric oxide, prostacyclin, soluble guanylate cyclase, adenylate cyclase, cardiovascular diseases, oxidative stress

## Abstract

The propensity towards platelet-rich thrombus formation increases substantially during normal ageing, and this trend is mediated by decreases in platelet responsiveness to the anti-aggregatory nitric oxide (NO) and prostacyclin (PGI_2_) pathways. The impairment of soluble guanylate cyclase and adenylate cyclase-based signalling that is associated with oxidative stress represents the major mechanism of this loss of anti-aggregatory reactivity. Platelet desensitization to these autacoids represents an adverse prognostic marker in patients with ischemic heart disease and may contribute to increased thrombo-embolic risk in patients with heart failure. Patients with platelet resistance to PGI_2_ also are unresponsive to ADP receptor antagonist therapy. Apart from ischemia, diabetes and aortic valve disease are also associated with impaired anti-aggregatory homeostasis. This review examines the association of impaired platelet cyclic nucleotide (i.e., cGMP and cAMP) signalling with the emerging evidence of thromboembolic risk in cardiovascular diseases, and discusses the potential therapeutic strategies targeting this abnormality.

## 1. Introduction

The hemostatic system consists of platelets, coagulation factors, and the endothelial cells lining the blood vessels. The regulation of hemostasis and vascular tone involves platelet–vessel wall interactions and platelet-derived vasoactive and pro-aggregatory substances. Platelet activation by ADP, TxA_2_, and serotonin results in platelet shape change, expression of surface receptors, adhesion, and aggregation, finally leading to thrombus formation. Under normal circumstances, the resistance of the endothelial cell lining to interactions with platelets and coagulation factors prevents thrombosis. When the endothelial continuity is disrupted due to vessel wall injury (e.g., due to atherosclerotic plaque rupture or erosion) and the underlying matrix is exposed, a coordinated series of events are set in motion to seal the defect (primary hemostasis) via rapid adhesion of platelets to the subendothelium. The initial hemostatic plug, composed primarily of platelets, is stabilized by a fibrin mesh that is generated in secondary hemostasis. Primary hemostatic disorders are categorized as impaired thrombus formation with associated prolongation of bleeding time due to functional disorders of platelets such as von Willebrand disease, Bernard-Soulier syndrome, and Glanzmann thrombasthenia [1].

On the other hand, an accelerated thrombus formation can occur due to platelet hyper-aggregability. While there is a balance between pro- and anti-aggregatory stimuli within the circulation, precisely tuned biochemical control over platelet function is paramount for normal hemostasis. However, the latter is often impaired in various cardiovascular disease states and, thus, contributes to an increased propensity towards thrombosis.

The induction of platelet aggregation by agents such as thrombin, collagen, TxA_2_, and adenosine diphosphate (ADP) reflects the activation, secondary to receptor binding of these agonists, of biochemical pro-aggregatory processes, notably the generation of inositol trisphosphate (IP_3_) and diacylglycerol (DAG). Furthermore, there is an increased transmembrane calcium influx, and an increased expression of P-selectin and of glycoprotein IIb/IIIa; platelet shape change precedes initiation of aggregation [2,3].

**Figure 1:**
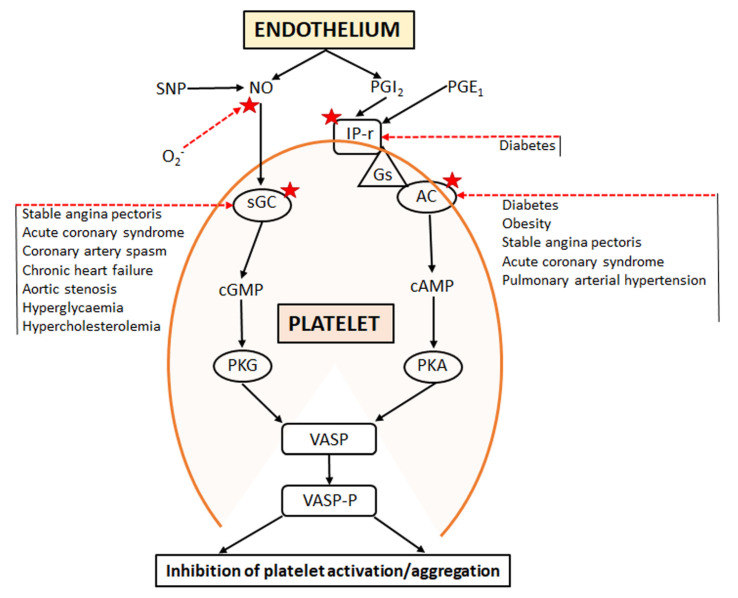
Schematic of endothelial/platelet interactions regarding GC/cGMP and AC/cAMP signalling and the impact of different disease states on anti-aggregatory responses to nitric oxide (NO) and prostacyclin (PGI_2_). Other abbreviations: AC = adenylate cyclase; Gs = stimulatory G-protein; IP-r = prostanoid IP receptor; PKA = cAMP-stimulated protein kinase; PKG = cGMP-stimulated protein kinase; SNP = sodium nitroprusside; sGC = soluble guanylate cyclase; VASP = vasodilator-stimulated phosphoprotein; VASP-P = phosphorylated VASP. Red stars define the sites of impaired signalling that are associated with different disease states and oxidative stress. The red arrows signify inhibitory impacts of various disease states: the black arrows imply progression through activation pathways.

Under physiological circumstances, a number of anti-aggregatory autacoids ensure a limitation on the net pro-aggregatory impact. Such autacoids are now known to include not only nitric oxide (NO) and prostacyclin (PGI_2_) (see Figure 1), but also hydrogen sulphide (H_2_S) and carbon monoxide (CO). NO, that is predominantly generated from arginine via NO synthases, diffuses across cellular membranes and activates the NO “receptor” soluble guanylate cyclase (sGC). The resultant generation of 3′5′-cyclic guanosine monophosphate (cGMP), in turn, induces phosphorylation of the vasodilator-stimulated phosphoprotein (VASP) and the inhibition of platelet aggregation [4].

PGI_2_ is generated indirectly from arachidonic acid and indirectly activates platelet adenylate cyclase (AC). Ultimately, this “cascade” also contributes to incremental VASP phosphorylation, a basis for potential synergy between NO and PGI_2_ anti-aggregatory effects [5], as shown in Figure 1.

In the context of cardiovascular disease states, the equilibrium between these pro- and anti-aggregatory forces is often skewed (whether through increased pro-aggregatory stimuli, decreased anti-aggregatory stimuli, or both), such that a heightened state of platelet activation (“platelet hyper-reactivity”) occurs. Indeed, platelet hyper-aggregability has been observed in both stable angina pectoris (SAP) and acute coronary syndromes (ACS) [6], atrial fibrillation (AF) [7,8], chronic (systolic) heart failure (CHF) [9], and coronary artery spasm (CAS) [10]. While increased pro-aggregatory stimuli may certainly contribute to platelet hyper-reactivity in these conditions, so too does the attenuation of function of the inhibitory pathways that are regulated by NO and PGI_2_. An impaired platelet response to NO has been documented in ACS, AF, and HF [11,12,13], as well as high cardiovascular risk conditions such as diabetes mellitus and obesity [14,15]. Similarly, there is emerging evidence [16] that the signalling pathway that is involved in platelet anti-aggregatory responses to PGI_2_ may also be impaired in patients with coronary heart disease (Figure 1).

The current review examines the association of impaired platelet cyclic nucleotide (i.e., cGMP and cAMP) signalling with the emerging evidence of thromboembolic risk in cardiovascular diseases and discusses potential therapeutic strategies that are targeting this abnormality.

## 2. Impairment of cGMP Signalling

NO is not only a fundamental modulator of vasomotor tone, but also a potent inhibitor of platelet aggregation [17]. NO, which is generated by endothelial NOS (eNOS), is an important component in preventing inappropriate thrombus formation, primarily via the activation of soluble guanylate cyclase (sGC). (Figure 1). Furthermore, NO generation is not only of endothelial origin, it is also released from activated platelets and, thus, provides a negative feedback to limit inappropriate platelet-rich thrombus formation [18,19,20].

The existence and/or functionality of platelet eNOS was, at one stage, a subject of controversy [21,22]. However, it is now established that human platelet subpopulations exist based on the presence and absence of eNOS [23]. Platelets that are lacking eNOS (approximately 20% of total platelets) fail to produce NO and have down-regulated cGMP-protein kinase (PKG) signalling. eNOS-negative platelets primarily initiate adhesion to collagen, more readily activate integrin αIIbβ3, secrete matrix metalloproteinase-2, and form larger aggregates than their eNOS-positive counterparts. Conversely, platelets that have an intact eNOS-sGC-PKG-signalling pathway (approximately 80% of total platelets) form the bulk of an aggregate and ultimately limit its size via NO generation.

**Figure 2:**
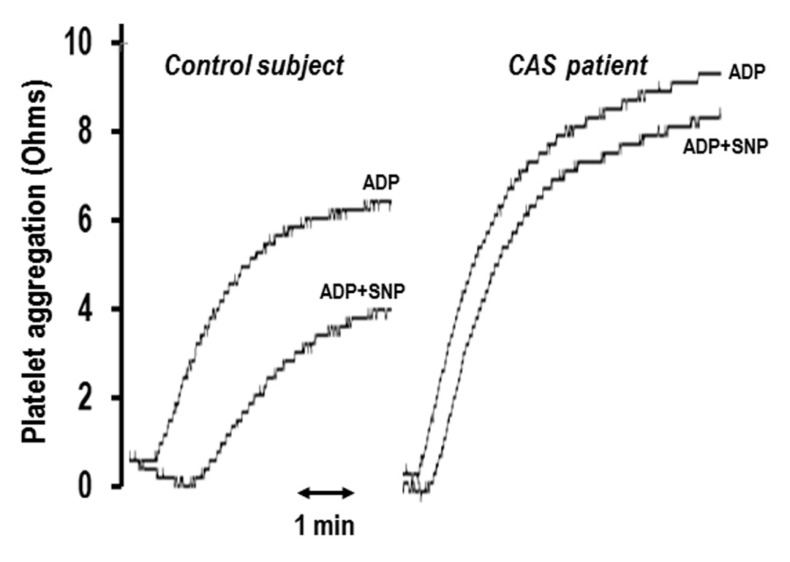
Representative aggregation tracings displaying both an increased platelet aggregation response to ADP (2.5 µM) and a diminished inhibitory effect of sodium nitroprusside (SNP, 10 µM) in a coronary artery spasm (CAS) patient (inhibition of 9%) comparative to control subject (inhibition of 33%). Modified with permission from Reference [10].

Importantly, platelets from patients with SAP, ACS, CAS, CHF, and also with risk factors for cardiovascular disease [10,11,12,24,25,26] exhibit reduced responsiveness to the anti-aggregatory effect of NO donors, a phenomenon that is termed “platelet NO resistance”. This is illustrated in Figure 2 which depicts the representative aggregation tracings displaying both an increased platelet aggregation response to ADP and a diminished inhibitory effect of the NO donor sodium nitroprusside (SNP, 10 µM) in a CAS patient compared to a healthy subject. NO resistance, whether documented at the vascular level [27,28] or with regards to platelet aggregation, is independently predictive of cardiovascular morbidity and mortality [28,29].

**Figure 3:**
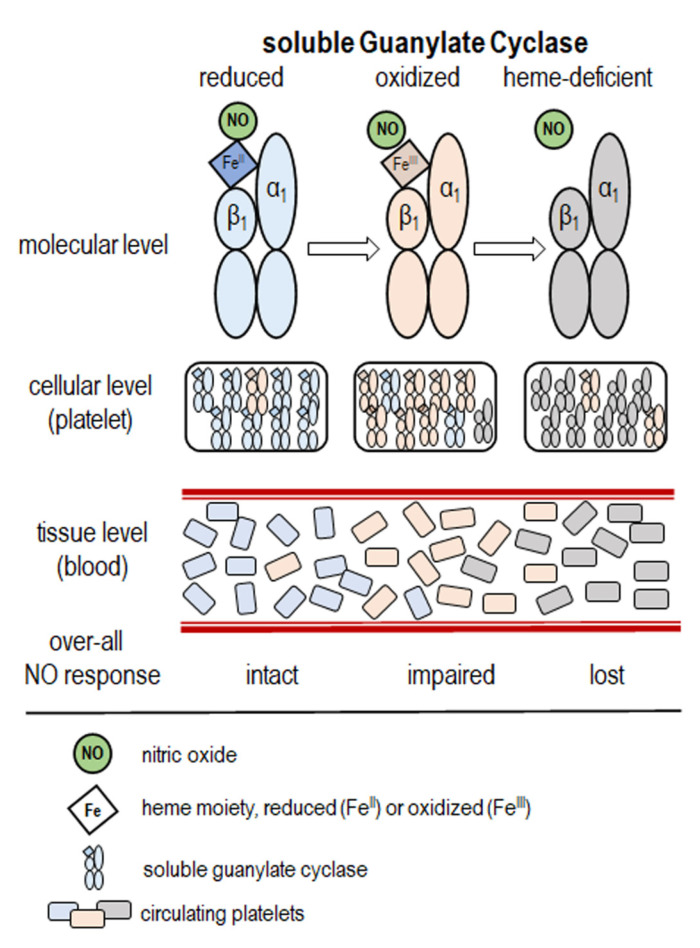
Bases for variability in platelet soluble guanylate cyclase (sGC) functionality. sGC is a heterodimer that is composed of one α and one heme-binding β subunit. In its FeII form, this heme moiety is the target of nitric oxide (NO), either endogenous (synthesized by endothelium) or exogenous. The binding of NO to the heme results in the activation of the catalytic domain, which produces cGMP from GTP. In conditions of oxidative stress, heme is oxidized to FeIII form, which has a lesser affinity for NO, resulting in diminished activation of sGC (“NO resistance”) and a loss of the heme moiety. Within platelets, there is a heterogeneity of the sGC molecular state (reduced, oxidized, and heme-deficient). The overall platelet responsiveness to NO in vivo reflects the relative prevalence of each of these sGC states.

Diminished sGC activation in response to NO has been documented in conditions of increased cardiovascular risk, such as hypercholesterolemia, diabetes, and polycystic ovarian syndrome [15,30,31,32,33], as well as in a number of cardiovascular disease states, including ACS, CHF, aortic valve stenosis, and newly diagnosed AF [11,12,13,34,35,36]. This was attributed to a reduction in platelet sGC sensitivity to NO, as schematized in Figure 3.

A number of post-translational modifications to the sGC enzyme may account for this:

(1) The oxidation/loss of the heme moiety, resulting in the impairment of sGC activation by NO [37];
(2) The oxidation/S-nitrosylation of the enzyme’s peripheral thiol moieties [38,39,40];
(3) A loss of expression of the constituent subunits of sGC, as was observed in a familial cohort exhibiting significantly increased incidence of myocardial infarction [41].

## 3. “NO Resistance” in Cardiovascular Disease States

### 3.1. Coronary Artery Disease

NO-sensitive sGC is the only NO receptor that is mediating platelet inhibition [42].

Impaired platelet NO-signalling has been identified in various disease states and conditions. Most attention so far has related to coronary artery diseases, especially ACS and SAP. In patients with SAP and ACS, as distinct from nonischemic patients and healthy subjects, platelet aggregability is increased, and the inhibition of aggregation by NO donors is decreased. Furthermore, multivariate analysis revealed that NO resistance occurs significantly more frequently with ACS than with SAP (odds ratio [OR] 2.3:1), where it less frequently [12,23,43]. The cardioprotective effects of some ACE inhibitors, such as ramipril (HOPE trial [44]) and perindopril (EUROPA study [45]) against recurrent ischemia may also reside, at least in part, in limitation of platelet NO resistance.

In ischemic patients, the suppressed anti-aggregatory effect of NO donors was paralleled by impaired sGC responsiveness to NO [46], engendered by increased oxidative stress that is usually associated with atherosclerosis. Atherosclerosis is a lipoprotein-driven disease that reflects lipid-rich plaque formation within the arterial tree through intimal inflammation, necrosis, fibrosis, and calcification. The disease usually remains silent for decades until suddenly causing life-threatening coronary thrombosis presenting as ACS (unstable angina pectoris or myocardial infarction). Most often, these cardiac emergences are associated with plaque rupture and exposure of thrombogenic, red cell-rich necrotic core material. Ruptures occur mainly among lesions that are defined as “thin-cap” fibro-atheroma. However, it has recently become apparent that thrombi forming on lesions without rupture (plaque/endothelial erosion) are also common, most often on sites of pathological intimal thickening [47].

Plaque rupture is caused by the loss of mechanical stability due to reduced tensile strength of the collagen cap surrounding the plaque. On the other hand, endothelial erosion, which is independent of plaque rupture, may occur after injurious insults to the endothelium that are instigated by metabolic disturbance or immune insults [48,49]. Post-mortem studies reported that plaque erosion is more frequent in young women [50]. Plaque erosion with an intact fibrous cap is now recognised to be responsible for about one third of ACS and up to two thirds of non-STEMI cases. It has been speculated that this epidemiological shift reflects changes in the patterns of plaque biology, perhaps with a decline in prevalence of “vulnerable plaques” as a result of the widespread use of statins [51].

Of note, patients with ACS that is caused by plaque erosion seem to have a better long-term prognosis compared to those with plaque rupture, and may be stabilized by dual antiplatelet therapy without the need for stenting, thus limiting the expense and potential complications of invasive procedures [52,53]. “Spontaneous” platelet aggregation and thrombosis can be impacted by shear stress, which has been considered as a possible driver for focal endothelial death and denudation. In parallel, neutrophil-dependent inflammation also promotes plaque erosion, possibly through a noxious action on endothelial cells [54].

Relatively little is currently known about the pathogenesis of “plaque erosion”. Indeed, the “plaques” that are involved in this process often contain relatively little lipid, and only minimal intra-plaque accumulation of macrophages, thus differing from the usual lesions that are involved in plaque rupture. Therefore, as might be expected, thrombi that are formed in association with plaque erosion tend to be platelet-rich.

The formation of neutrophil extracellular traps (NETs) promotes endothelial cell activation and increased thrombogenicity through the concerted action of IL-1α and cathepsin G. Erosion-prone plaques associate with more NETs than lesions with stable or rupture-prone characteristics. Thus, NETs may amplify and propagate endothelial dysfunction that is related to thrombosis, due to superficial erosion [55,56,57].

Plasma myeloperoxidase (MPO) concentrations are significantly higher in patients with plaque erosion versus plaque rupture (median (interquartile range), 96.3 ng/mL (44.2–173.3) vs. 41.7 ng/mL (29.2–66.3); *p* < 0.001). Multivariable logistic regression analysis indicated that plasma MPO was independently associated with plaque erosion (odds ratio, 3.25; 95% confidence interval, 1.37–7.76; *p* = 0.008) [58].

These findings raise the possibility that “plaque erosion” is a process that is primarily related to inflammatory damage to the endothelial glycocalyx (“glycocalyx shedding”) [59], rendering the vascular endothelium more prone towards platelet-rich thrombus formation. Furthermore, they tend to implicate hypochlorous acid, which is generated by myeloperoxidase, as a mediator of this erosive process.

### 3.2. Coronary Artery Spasm

It is increasingly recognised that CAS plays a substantial role in the pathogenesis of reversible myocardial ischemia, but as an isolated form of coronary vasomotor pathophysiology and also in combination with large coronary artery atheromatous disease. Furthermore, many studies have shown that both platelet activation and platelet-rich thrombus formation occur frequently in patients with CAS. Nevertheless, the pathophysiology of CAS, whether on a large or small coronary artery basis, with its associated ischemic crises, is currently poorly understood and treatment is frequently ineffective. CAS is characterised by extreme NO resistance at the platelet level and substantial impairment of platelet NO signalling. There is increasing evidence that CAS symptomatic crises are precipitated by the impairment of NO/H_2_S signalling, resulting in combined vasoconstriction and increased platelet aggregation. Treatment of patients with high-dose N-acetylcysteine (NAC) plus low-dose nitroglycerin (NTG) rapidly increased platelet responsiveness to the NO donor sodium nitroprusside (SNP). This effect of NAC, a metabolic precursor of H_2_S, was confirmed in vitro and mimicked by the H_2_S donor NaHS consistent with the known potentiation of NO effects by H_2_S. Conversely, the inhibition of enzymatic production of H_2_S attenuated the NAC effect. There is now strong evidence that CAS crises involve acute damage to the vascular endothelium, with erosion of its inner glycocalyx layer, together with the activation of platelet aggregation, and with resultant macro-or micro-thrombosis in the absence of plaque rupture [10,60]. NAC, via the release of H_2_S, reverses platelet resistance to NO and terminates glycocalyx shedding during symptomatic crises. This suggests that H_2_S donors may correct the pathophysiological anomalies that are underlying CAS and other cardio-vascular disease states [61].

### 3.3. Heart Failure

CHF, a condition that is also characterized by a prothrombotic state and enhanced oxidative stress [62], provides another example of platelet resistance to NO [11,63,64,65]. The substantial clinical evidence that CHF represents a prothrombotic disorder includes post-mortem studies showing that many such patients die of coronary thromboses [66]. Furthermore, the presence of CHF increases the thrombo-embolic risk among patients with chronic atrial fibrillation, regardless of the mode of anticoagulation [67]. Similarly, the platelet and vascular responses to exogenous NO donors are impaired in CHF patients compared with healthy volunteers, thus limiting the therapeutic potential of NO donors both as afterload-reducing and anti-aggregatory agents in this setting.

### 3.4. Aortic Valve Stenosis

Aortic valve stenosis (AS) occurs frequently in ageing populations and represents a common basis for the need for valve surgery. Interestingly, even the earliest phases of AS (“aortic sclerosis”) represent independent predictors of the risk of acute myocardial infarction. It turns out that both aortic sclerosis and AS are associated with substantial platelet hyperaggregability and the impairment of anti-aggregatory responses to NO. Thus, AS is a clinical marker of NO resistance and of coronary risk. This is not particularly surprising given that NO also functions to retard the development of aortic valve calcification [68,69,70,71].

### 3.5. Diabetes

Diabetes is associated with accelerated atherogenesis and a high incidence of cardiovascular events. Patients with Type 2 diabetes mellitus (T2DM) exhibit reductions in NO bioavailability and NO responsiveness in the vasculature, platelets, and myocardium [72]. It has been shown recently [73] that SNP and NTG inhibit ADP-induced aggregation in T2DM to a lesser extent than in control subjects. The abnormal platelet function in diabetics is partly mediated by an increased blood glucose level [74]. Indeed, the risk of thrombus formation is increased in diabetics with poor glycaemic control [75]. On the other hand, normalization of platelet aggregability was associated with acutely improved glycaemic control [76]. A direct correlation exists between platelet hyperaggregability and plasma glucose in patients with acute myocardial infarction [77]. Furthermore, in diabetics with ACS, an inverse correlation has been documented between admission blood-sugar level and platelet SNP responsiveness, and a positive correlation with whole blood O_2_^−^ levels [15].

Under chronic conditions, it is likely, but not absolutely proven, that platelet resistance to NO and to PGI_2_ is exaggerated in the presence of concomitant obesity [78]. An important, but currently unanswered, question is to the extent that insulin resistance in T2D modulates responsiveness to NO.

### 3.6. Aging

Inflammation is an important contributor towards the progression of many forms of cardiovascular disease.

A number of recent studies have demonstrated that the aging process is closely associated with extensive inflammatory activation and this inflammation, in turn, contributes to age-related increases in incidence of many forms of cardiovascular and cerebrovascular disease [69,79].

Platelet NO resistance occurs increasingly with advanced age and its extent is directly proportional to platelet expression of the NLRP3 inflammasome activator thioredoxin-interacting protein. While the impaired generation and signalling of NO contributes substantially to cardiovascular risk that is associated with hypertension, hyperlipidemia, and diabetes mellitus, advanced age is, in itself, a consistent and independent cardiovascular risk factor. Many processes that are involved in aging are modulated by NO and aging is associated both with impaired NO signalling and with increases in plasma concentrations of the endogenous NO synthase inhibitor, asymmetric dimethylarginine (ADMA). There is a significant correlation between changes in these parameters over time. Following multivariate analyses, independent correlates of a decline in platelet responsiveness to NO with increasing age are female gender and low vitamin D concentrations, whereas increases in ADMA were associated with the presence of diabetes and impaired renal function [80].

It has recently been demonstrated that COVID-19 survivors exhibited a significant acceleration of their biological ageing and telomere shortening, occurring mainly in the younger individuals [81]. Additionally, the expression of the angiotensin-converting enzyme 2 (ACE2, which hydrolyses angiotensin II to angiotensin (1-7) and counteracts the ACE effects in the vascular system) was decreased in post-COVID-19 patients, compared with the COVID-19-free population [82]. This may mediate the prothrombotic phenotype in COVID-19, with many patients developing acute cardiac injury during the course of the illness [83] and increased an incidence of thrombotic complications [84].

Thus, ageing and its contributors or molecular mediators might furnish targets for novel therapeutic strategies that could promote healthy ageing and conserve resources for health care systems worldwide.

## 4. Mechanisms of NO Resistance

The classical view of “endothelial dysfunction” represents its pathogenesis as an ill-defined combination of decreased NO formation and impaired NO effect. It turns out that there are several potential causes of reduced NO effect (“NO resistance”), including accelerated clearance of NO (often via combination with superoxide anion), impaired activity of the NO receptor sGC, and the accelerated clearance of cGMP, the active product of sGC, by a variety of phosphodiesterases.

Soluble GC, the mammalian NO sensor and principal receptor, transduces the NO signal to the second messenger cGMP. NO binds to the ferrous (Fe^2+^) oxidation state of the sGC heme cofactor and accelerates formation of cGMP several hundred-fold. Oxidation of the sGC heme to the ferric (Fe^3+^) state desensitizes the enzyme to NO and even causes heme loss. For example, the proportion of heme-free enzyme in rat platelets increased distinctly under conditions of heme oxidation due to increased spontaneous heme-loss [85] Furthermore, sGC undergoes a reductive nitrosylation reaction that is coupled to the S-nitrosation of peripheral sGC cysteines, and heme-assisted nitrosothiol formation of β1Cys-78 and β1Cys-122 causes the NO desensitization of ferric sGC. Thus, reductive nitrosylation is gated by a conformational change of the protein and determines function and dysfunction of sGC in cardiovascular disease [86].

The impairment in sGC function also can be caused by increased O_2_^−^. Indeed, O_2_^−^ inhibits human platelet guanylate cyclase [87] and enhances platelet aggregation both in vitro [88] and in the animal model in vivo [89]. Of note, many forms of acute and chronic cardiovascular disease, which results in increased levels of oxidative stress, are accompanied by a reduced expression of sGC in the vessel wall [90,91]. It would be good to know whether the same applies to platelets.

In theory, accelerated catabolism of cGMP by 3′5′-cyclic nucleotide phosphodiesterases might also contribute to NO resistance. However, in practice, the extent of platelet NO resistance is independent of the integrity of such phosphodiesterases [43].

Similarly, it is possible that the impaired activation of cGMP-specific protein kinases might be involved in NO resistance [92]. However, platelets from patients with NO resistance, although hyporesponsive to NO donors, retain normal anti-aggregatory responsiveness to dibutyryl-cGMP. Thus there is no evidence that NO resistance is mediated by this distal step in the NO/sGC “cascade” [93].

As mentioned above, platelets express eNOS [18,94], an enzyme that is responsible for the generation of NO, which provides a negative feedback on platelet activation and aggregation. Impaired platelet production of NO is associated with thrombosis and ACS [18]. However, decreased formation of NO, by definition, does not contribute to NO resistance. As to whether lack of eNOS cofactor activity contributes to decreases in NO formation in platelets, this certainly remains a substantial possibility, which, thus far, has been validated especially in vascular smooth muscle. Furthermore, NO may be generated by enzymatic reduction of nitrite anion, and this process is also relevant to the control of platelet aggregability. The extent to which variable nitrite reduction compromises anti-aggregatory homeostasis has not yet been fully investigated [95,96].

A decrease in the anti-aggregatory effect of NO also could be due to the increased clearance of NO by superoxide anion-radical O_2_^−^ (Figure 1). The generation of O_2_^−^ is augmented in oxidative stress, which is associated with many cardiovascular disease states [11,18,97,98]. In our studies, patients with SAP had four-fold higher blood levels of O_2_^−^ as compared with normal subjects [43]. Nicotinamide adenine dinucleotide phosphate (NADPH) oxidase, which catalyzes the reduction of molecular oxygen to O_2_^−^, is a key enzyme in the pathobiology of oxidative stress. Angiotensin (Ang) II is an important activator of this enzyme [99,100]. This raises the question of whether Ang II augments NO resistance. On the other hand, angiotensin-(1-7) [Ang-(1-7)], a heptapeptide that is generated by ACE2, has been proposed as a physiological antagonist of Ang II [101,102], and it potentiates the anti-aggregatory action of SNP [103]. Therefore, Ang-(1-7) may counteract platelet NO resistance. As discussed above, the decreased generation of Ang-(1-7) has been implicated in the acceleration of vascular aging during recovery from COVID-19 infection.

Thus, the bioavailability of NO and the integrity of sGC are crucial for an adequate platelet response to exogenous NO, while eNOS expression and sGC activity represent key modulators of endogenous NO signalling.

### 4.1. Prognostic Implications of NO Resistance

NO resistance, both at the vascular and platelet levels, is an independent marker of poor prognosis [28,104,105]. Additionally, and unsurprisingly, NO resistance has been linked with endothelial dysfunction [106,107]. For example, it was demonstrated that, in patients that were at risk for atherosclerosis, reduced endothelium-dependent vasodilatation was directly correlated with impaired NTG-related dilatation [106]. Analogously, platelet NO resistance may also be a prognostic tool. In a cohort of patients with ACS, we have demonstrated that the extent of platelet NO resistance is associated with adverse outcomes [29]. Specifically, patients with platelet NO resistance at initial presentation had higher cardiovascular morbidity and mortality at the seven-year follow-up in comparison with patients who had preserved platelet NO responsiveness at first presentation. Thus, the identification of patients with platelet NO resistance might constitute a means for improved therapeutic decision-making. The development of such strategies is contingent on the demonstration of an interconnection between the amelioration of NO resistance and improved outcomes.

### 4.2. Circumvention and Amelioration of Platelet NO Resistance

NO resistance can be regarded as a potential target for pharmacological interventions that are designed either to enhance platelet NO responsiveness and/or activate sGC in an NO-independent manner. There are two possible strategies in this regard that are the circumvention of platelet NO resistance (for example by NO-independent stimulators or activators of sGC) or its therapeutic amelioration (via targeting the extracellular environment and conditions such as oxidative stress or a deficiency in essential factors for NOS/NO functionality).

### 4.3. Circumvention of NO Resistance

#### 4.3.1. Nitrite (NO_2_^−^)

Furthermore, nitrite increases intra-platelet levels of cGMP and the phosphorylation of VASP at Ser239 in the presence of partially deoxygenated erythrocytes [108]. This is of considerable clinical interest: the concept of being able to deliver a vasodilator and anti-aggregatory stimulus selectively to hypoxic regions has major potential clinical applications. In view of that, the inhibition of ADP-induced platelet aggregation by NO_2_^−^, in comparison with that of SNP was assessed both in whole blood samples and platelet-rich plasma (PRP) [109].

Evidence that NO_2_^−^ -related anti-aggregatory effects are mediated by the activation of sGC was also strengthened by the fact that there was a strong correlation between NO_2_^−^ and SNP effects, and that NO_2_^−^ effects were also subject to NO resistance. However, effects of NO_2_^−^ were greater in venous than in arterial blood, and were potentiated by hypoxia. Overall, it might be suggested that NO_2_^−^ represents a practicable means for preserving sGC activation under hypoxia, or within the venous circulation, but strictly speaking, it does not circumvent NO resistance.

On the other hand, a recent study reported that nitrite can at least partially circumvent platelet NO resistance independently of other blood cells by directly activating sGC and phosphorylating VASP [64]. The study assessed collagen-induced aggregation in washed platelets that were obtained from HF patients with reduced ejection fraction in comparison with healthy subjects. While the inhibition of platelet aggregation by the NO donor SNP was impaired in patients, due to sGC dysfunction there was no diminution of the anti-aggregatory effects of nitrite. Given that, in a washed platelets preparation, there are no red blood cells (or deoxyhemoglobin) to facilitate a release of NO from nitrite, the authors speculated that nitrite might have been acting as a direct NO-independent sGC activator, in analogy with Bay 41-2272. However, there is a misconception here. Bay 41-2272, by definition, is not an “activator”, but a “stimulator” of sGC [37,110], which means that, while this compound just potentiates responses of sGC to NO, it does not bind directly to sGC; the biochemical effect of a “stimulator” is revealed only with NO being bound to the heme moiety of sGC. Therefore, the authors’ premise appears unsubstantiated. Even if the authors were referring to a true “activator” (such as Bay 58-2667), the direct binding of nitrite to the un-occupied heme pocket on the sGC molecule is unlikely.

In conclusion, while potential clinical utility of nitrite is not addressed by these experiments, its relevance to the circumvention of NO resistance requires further clarification.

#### 4.3.2. Nitroxyl

Nitroxyl is an NO “sibling” that is capable of activating sGC [111,112]. In vivo, nitroxyl is thought to exist in its protonated form (HNO). It has no unpaired electron, and because of that, is resistant to scavenging by O_2_^−^ under conditions of increased O_2_^−^ release. Nitroxyl, similar to NO, produces vasodilator and anti-aggregatory effects, largely via sGC activation Therefore, HNO donors might be expected to induce continued sGC stimulation despite oxidative stress and, thus, circumvent NO resistance. With a suite of vasoprotective properties and an ability to enhance cardiac inotropic and lusitropic responses, coupled with preserved efficacy in the setting of oxidative stress, HNO donors have intact therapeutic potential in the face of diminished NO signalling [72].

In a previous in vitro study [107], the inhibition of platelet aggregation by the HNO donor isopropylamine NONOate (IPA/NO) and the NO donor SNP were compared in subjects and patients with a wide range of responses to NO. Overall, SNP (10 μM) induced 30% inhibition of platelet aggregation, and IPA/NO (10 μM) caused 75% inhibition. Importantly, in NO-resistant subjects, the IPA/NO:SNP response ratio was markedly increased, and cGMP accumulation in platelets was greater with IPA/NO than with SNP stimulation. The heme-dependent inhibitor of sGC ODQ attenuated responses to both SNP and IPA/NO, although the extent of this inhibition was significantly greater for SNP (*p* < 0.03). Thus, the HNO donor IPA/NO substantially, but not entirely, circumvents platelet NO resistance while acting, at least partially, as a heme-mediated sGC activator.

### 4.4. NO-Independent Stimulators and Activators of Guanylate Cyclase

From a functional point of view, it is critically important that sGC has a closely associated heme group which plays a major part in the stimulation of enzymatic activity by NO. The oxidation of sGC under redox stress leads to loss of the heme group, with a dramatic loss of responsiveness to NO.

Within the last 20 years, a number of novel compounds have been synthesized which are capable of NO-independent activation of oxidized or heme-deficient sGC. These compounds can be divided into heme-dependent “sGC stimulators”, with a classical example of riociguat (BAY 41-2272), and heme-independent “sGC activators”, for example cinaciguat (BAY 58-2667). They are potent vasorelaxants, inhibit platelet aggregation and decrease blood pressure under hypertensive conditions [113,114], and also are valuable tools to elucidate the physiology and pathophysiology of the NO/sGC signalling pathway in more detail [115].

The sGC stimulator riociguat is on the market and is approved for the treatment of pulmonary hypertension, and a variety of other sGC stimulators are in preclinical and clinical development for additional indications [110]. Also, the recently published results of the VICTORIA trial [116] show that the sGC stimulator vericiguat provides symptomatic improvement in patients with CHF. sGC stimulators sensitize sGC to NO by stabilizing NO–sGC binding and they are also able to directly stimulate the native form of the enzyme independently of NO [117].

In contrast to the heme-dependent sGC stimulators which strongly synergize with NO, sGC activators interact with heme-free sGC, which is totally unresponsive to NO.

In particular, the additive effect of Bay 58-2667 and NO-donors entirely originated from the addition of the respective catalytic rates [37]. The activation of sGC by Bay 58-2667 results in anti-aggregatory activity, both in vitro and in vivo, and induces dose-dependent increases in the intra-platelet cGMP content. In human platelet-rich plasma, Bay 58-2667 inhibited platelet aggregation that is induced by ADP, collagen, and the TxA_2_ mimetic U-46619. In animal studies employing the FeCl_3_ thrombosis model, Bay 58-2667 prolonged bleeding time and reduced thrombus formation.

Clinical development of this group of drugs remains limited to date, and their potential for inducing a prothrombotic state via the excessive activation of sGC might represent a problem, at least in theory. Certainly, they should be regarded not only as vasodilators, but also as anti-aggregatory agents from a clinical point of view.

### 4.5. Amelioration of Platelet NO Resistance

#### 4.5.1. Angiotensin-Converting Enzyme (ACE) Inhibitors

Some ACE inhibitors (ramipril and perindopril) have been shown to reduce the risk of coronary events in patients with T2DM and/or previous myocardial ischemia. Furthermore, all ACE inhibitors have been shown to improve symptomatic status and prognosis in patients with systolic heart failure [118,119]. In patients with CHF who are not treated with ACE inhibitors, platelets exhibit hyperaggregability and impaired responsiveness to the anti-aggregatory and cGMP-stimulatory effects of SNP, as compared to healthy subjects; increased blood levels of O_2_^−^ parallel this. The initiation of perindopril therapy in these patients was associated with an increase in platelet responsiveness to anti-aggregating and cGMP-stimulating effects of SNP and a decrease in the whole blood O_2_^−^ content [11,63]. Furthermore, in a high-risk (“HOPE study”-type) patient cohort, three-months’ therapy with ramipril significantly increased platelet SNP responsiveness relative to the placebo [65].

There was also some evidence from this study to support the concept that the potentiation of platelet response to NO might be greater in patients with prior NO resistance than in patients with initially normal responses. Therefore, it seems not only likely that the normalization of anti-aggregatory homeostasis plays a role in the cardioprotective effects of Ramipril in high-risk patients, but also that there is little risk of ‘overshoot” of these effects to increased bleeding rates.

#### 4.5.2. Angiotensin-(1-7)

In whole blood samples that were obtained from patients with ACS and healthy controls, angiotensin (Ang) II augmented U46619-induced platelet aggregation, while Ang-(1-7), a product of ACE2 activation, did not directly affect platelet aggregation but potentiated the anti-aggregatory action of SNP [103]. Therefore, Ang-(1-7) ameliorates platelet NO resistance. Furthermore, in blood samples that were obtained from healthy subjects, ADP-induced platelet aggregation was accompanied by O_2_^−^ release, and SNP not only inhibited platelet aggregation but also suppressed this O_2_^−^ release; Ang-(1-7) augmented these effects of SNP [120]. Importantly, the Ang-(1-7) effects took place only in whole blood, not in the platelet-rich plasma. Furthermore, the Ang-(1-7) receptor antagonist D-ala7-Ang-(1-7) eliminated the potentiating effects of Ang-(1-7). Thus, Ang-(1-7) can ameliorate platelet NO resistance, presumably via a suppression of O_2_^−^ release occurring during the aggregation as a result of an interaction between platelets and neutrophils.

Due to the fact that Ang-(1-7) heptapeptide is also metabolized by ACE, circulating levels of Ang-(1-7) increase up to 25-fold during ACE inhibition [121], and this could contribute to the beneficial effect of ACE inhibitors on the amelioration of NO resistance. As mentioned above, the expression of ACE2 is decreased in post-COVID-19 patients, with the consequent decrease in Ang-(1-7) concentration in blood [82] and an increased incidence of thrombotic complications [84]. The administration of ACE inhibitors would be beneficial in these conditions as well.

In summary, these findings suggest that Ang-(1-7) mediates some of the anti-ischemic effects of ACE inhibitors. Pharmacological strategies to increase Ang-(1-7) blood levels may have advantageous effects both on platelet and vascular homeostasis.

#### 4.5.3. Statins

Hypercholesterolemia represents the best-documented risk factor that is associated with endothelial dysfunction. Statins, in general, ameliorate endothelial dysfunction [122,123,124], however, there is insufficient information about their impact on vascular and platelet NO responsiveness. In our study involving patients with ACS [12], the results of multivariate analysis showed that NO resistance was less common among patients that were treated with statins (odds ratio 0.45:1). In a later study [33], treatment with pravastatin (40 mg/day for 3 months) improved platelet NO responsiveness and increased NO bioavailability in asymptomatic subjects with and without mild hyper-cholesterolemia. In addition, there was an inverse relationship between the change in superoxide levels and change in SNP responsiveness.

Thus, a potential beneficial effect of statin therapy on platelet NO responsiveness is promising but requires further elucidation.

#### 4.5.4. Perhexiline

Perhexiline is an effective prophylactic anti-anginal agent which improves myocardial energetics [125] and is utilized in the management of patients with refractory angina in whom coronary revascularization is impractical [126]. Perhexiline also improved the hemodynamic and functional status in patients with CHF [127] and SAP [128]. Perhexiline also appears to exert anti-inflammatory effects, primarily via the inhibition of NAD(P)H oxidase [129]. We have demonstrated in patients with ACS [130], that therapy with perhexiline for three days was associated with increases in SNP-induced inhibition of platelet aggregation. Importantly, the resolution of symptomatic ischemia was associated with significantly greater increases in platelet SNP responsiveness than non-resolution. An increase in platelet SNP responsiveness also occurred after perhexiline therapy in patients with SAP and was associated with an 85% decrease in anginal frequency. Treatment with perhexiline potentiated the ex vivo cGMP-elevating effects of SNP in platelets. Perhexiline also inhibited O_2_^−^ release from neutrophils in vitro. Whether the observed potentiation of NO effects by perhexiline could be regarded as fundamental to its beneficial impact on platelet NO responsiveness remains to be established.

#### 4.5.5. Glycaemic Control

In vivo and in vitro studies have demonstrated that hyperglycaemia directly induces endothelial dysfunction and attenuates endothelium-dependent vasorelaxation [131,132]. Furthermore, high glucose concentrations decrease platelet NO production [133]. However, insulin infusion in humans inhibits ex vivo platelet aggregation (in response to collagen, thrombin receptor-activating peptide, ADP, and epinephrine [134]) partially via the activation of platelet NO synthase [135]. In diabetics that were admitted with ACS, increased blood sugar levels were directly correlated with blood levels of O_2_^−^ [15]. In these patients, platelet responsiveness to the antiaggregatory effects of the NO donor SNP was impaired. However, intravenous insulin infusion for 12 h resulting in a significant reduction in the blood sugar levels improved platelet responsiveness to SNP and decreased blood levels of O_2_^−^. These findings provide a further rationale for the utilization of insulin therapy in diabetic hyperglycaemic patients with ACS.

## 5. Impairment of cAMP Signalling

In platelets, cAMP is generated by adenylate cyclase (AC), which exists predominantly as an AC3 transmembrane isoform linked to G-protein-coupled receptors [136]. However, AC5 and AC6 isoforms have also been reported [137]. AC activation occurs in microdomains within the platelet membrane (localized to “lipid rafts”) [138], so that changes in AC may not parallel the whole cell distribution of generated cAMP. The physiological effects of AC/cAMP signalling in platelets reflect interactions of membrane-bound AC molecule with stimulatory (Gs) or inhibitory (Gi) G-proteins; the integrity of both is paramount for adequate platelet response to external stimuli. Therapeutic interest in AC-based signalling in platelets has been centered on the role of prostacyclin (PGI_2_), acting via IP receptors [139].

PGI_2_ is the most potent natural inhibitor of platelet aggregation and is found in most mammals that use platelet-mediated thrombosis for blood clotting. It plays an important role in maintaining cardiovascular health, particularly in preventing thrombotic occlusions in blood vessels. However, the activation of AC may also occur via stimulation of other cell surface receptors (e.g., beta-adrenoreceptors and/or adenosine receptor A2A). Furthermore, forskolin and its analogues can activate AC directly, independent of cell surface receptors [140].

Early studies [141,142] have reported that platelets from diabetic patients exhibited diminished sensitivity to PGI_2_ (and its analogues), which correlated with decreased cAMP generation. Obese patients have also displayed diminished platelet PGI_2_ response [14,143]. PGE_1_, which also can engage the IP receptor, produced a concentration-dependent increase in intraplatelet cAMP and there was a strong correlation between the cAMP-stimulating and antiaggregating effects of PGE_1_. However, platelet responsiveness to anti-aggregatory prostanoids that activate AC varies in patients that are prone to the development of coronary thrombosis. Specifically, patients with SAP exhibited platelet hypo-responsiveness to the antiaggregatory effect of PGE_1_, and diminution in cAMP generation, comparative to healthy subjects [16]. These in vitro results are likely also to imply reduced platelet sensitivity in vivo to endogenous PGE_1_ and PGI_2_, which, in turn, might contribute to the platelet hyperaggregability that is observed in cardiovascular diseases.

Another example of PGI_2_ resistance is idiopathic pulmonary arterial hypertension (IPAH). Even though most of the pathology of IPAH is observed in the lung, there is systemic involvement. In patients with IPAH, platelets show abnormalities in the PGI_2_ and NO pathways. A reduced expression of the G protein αs (stimulatory) subunit and an increased expression of the “regulatory” (suppressive) subunits of the cAMP-dependent protein kinase were detected, aggravating an overall decrease in the activation of the prostacyclin pathway [144]. Interestingly, these platelets exhibited reduced levels of the sGC subunits and increased the expression of the phosphodiesterase type 5A, conditions that disrupt the response to NO.

A recent study examined platelet function in patients with T2DM compared to healthy age-matched controls in whom hypoglycaemia was induced [145]. Hyper-insulinaemic clamps reduced the blood glucose to 2.8 mmol/L (50 mg/dL) for one hour. Sampling at baseline; euglycaemia 5 mmol/L (90 mg/dL); hypoglycaemia; and at 24 h post clamps were undertaken. The ability of PGI_2_ to inhibit platelet activation (inhibition of fibrinogen binding and P-selectin expression) was significantly impaired at 24 h following hypoglycaemia compared to the baseline in the T2DM group. Thus, impaired platelet sensitivity to PGI_2_ can engender platelet hyperactivity.

A major recent stimulus to investigations of the variability in platelet AC signalling has been provided by the clinical development of ADP antagonists, which interact with the P2Y_12_ receptor, suppressing platelet aggregation [146]. These include the thienopyridine derivatives clopidogrel and prasugrel, which are pro-drugs, and ticagrelor, which directly and reversibly inhibits P2Y_12_ [147]. P2Y_12_ receptor is coupled to the inhibitory G-protein Gi2α. The engagement of this G-protein inhibits AC and reverses the stimulant effects of PGI_2_ on AC, with the consequent suppression of the anti-aggregatory effects of cAMP pathway [148].

The impaired responsiveness to PGI_2_, as measured by the attenuation of adenylate cyclase stimulation, is now recognized as a mechanism for homeostatic impairment at the platelet level. It is now of considerable importance to determine whether this is a consequence of normal aging, given recent evidence that low dose aspirin, which acts predominantly to alter the spectrum of prostanoid signalling towards PGI_2_, has proved disappointing in elderly individuals.

However, of the greatest current interest is the finding of a spectrum of variability in the coupling of the P2Y_12_ receptor with inhibitory G-proteins [147]. Since a blockade of the P2Y_12_ receptor would reverse ADP-induced inhibition of platelet AC, the efficacy of ADP antagonists in individual patients could be predicted by pre-treatment AC responses to PGI_2_ (or PGE_1_) [148,149]. Indeed, for both acute and subacute responses, a close association emerged which, in the case of weight-adjusted subacute therapy, exceeded the importance of clopidogrel activator genotype. We were able to determine, via correlation between the responses to PGE_1_ and those to the direct AC stimulator forskolin, that the dysfunction of AC is a major contributor to platelet resistance to ADP antagonists [148]. It is quite likely that the impairment of the nexus between the P2Y_12_ receptor activation and the integrity of the PGI_2_/AC axis represents the basis for the “North American Paradox”, whereby high dose aspirin therapy is associated with a diminution of the effectiveness of ticagrelor [150,151].

## 6. Conclusions and Future Perspectives

Platelet resistance to anti-aggregatory effects of NO donors is frequently present in patients with cardiovascular disease states (Figure 2). NO resistance results largely from a combination of “scavenging” of NO by O_2_^−^ and of the inactivation of sGC (Figure 1). The prognostic impact of NO resistance has been demonstrated in patients with ischemic heart disease and it has been shown that several agents (ACE inhibitors, perhexiline, insulin, and statins) ameliorate this anomaly.

NO resistance has been also documented in vasculature and is usually paralleled by endothelial dysfunction. The concept that redox stress-induced impairment of anti-aggregatory efficacy at the platelet level is paralleled by an increasing interest in the possibility that both anatomically-based endothelial erosion (“glycocalyx shedding”) and impaired vascular responsiveness to vasodilator autacoids such as NO is of increasing clinical interest, especially secondary to recent reports that both coronary artery spasm and acute myocardial infarction often reflect abnormal vascular/platelet interactions.

The impairment of cAMP signalling was documented in several cardiac disease states and revealed a decreased platelet responsiveness to anti-aggregatory effects of PGI_2_ and PGE_1_. While in some cases it goes in parallel with the impairment of cGMP signalling, this is not a generality. In some conditions (for example, CAS), cAMP signalling is not as badly affected as cGMP signalling. However, in cardiac patients with diabetes it is selectively affected. This likelihood that the extent of impairment of platelet responsiveness to the anti-aggregatory effects of NO and of PGI_2_ (and indeed of other anti-aggregatory autacoids) may be widely disparate in many thrombotic risk states is also likely to limit individualization and titration of patients’ treatments on the basis of baseline and serial assessment of platelet responsiveness to individual autacoids. Nevertheless, it would be desirable to determine whether the extent of improvement of platelet responsiveness to NO (a validated marker of impaired prognosis) under pharmacotherapy predicts the extent of improvement of prognosis. If that were the case, routine “on-treatment” evaluation of responsiveness to NO would be justified.

With regards to the pharmacological improvement of platelet responsiveness to anti-aggregatory prostanoids, little effort has been so far made to circumvent or ameliorate such a dysfunction, despite the considerable physiological and therapeutic significance of AC signalling.

## Data Availability

Not applicable.
